# Clinical Accuracy of Large Language Models and Google Search Responses to Postpartum Depression Questions: Cross-Sectional Study

**DOI:** 10.2196/49240

**Published:** 2023-09-11

**Authors:** Emre Sezgin, Faraaz Chekeni, Jennifer Lee, Sarah Keim

**Affiliations:** 1 Nationwide Children's Hospital Columbus, OH United States; 2 College of Medicine The Ohio State University Columbus, OH United States; 3 College of Public Health The Ohio State University Columbus, OH United States

**Keywords:** mental health, postpartum depression, health information seeking, large language model, GPT, LaMDA, Google, ChatGPT, artificial intelligence, natural language processing, generative AI, depression, cross-sectional study, clinical accuracy

## Introduction

Postpartum depression (PPD) affects about 1 in 8 women in the months after delivery [1], and most of the affected individuals do not receive help, primarily due to insufficient screening and a lack of awareness about the condition. As large language model (LLM)–supported applications are becoming an integral part of web-based information-seeking behavior, it is necessary to assess the capability and validity of these applications in addressing prevalent mental health conditions [2]. In this study, we assessed the quality of LLM-generated responses to frequently asked PPD questions based on clinical accuracy (a contextually appropriate response that reflects current medical knowledge).

## Methods

We used 2 publicly accessible LLMs, GPT-4 (using ChatGPT) [3] and LaMDA (using Bard) [4], and Google Search engine. On April 3, 2023, we prompted each model and queried Google with 14 PPD-related patient-focused frequently asked questions sourced from the American College of Obstetricians and Gynecologists (ACOG; Multimedia Appendix 1) [5]. ChatGPT and Bard were prompted with each question in a new single session without prior conversation. Google Search results were not standardized, and search results were displayed in 3 different formats: an information card, curated content (a snippet of text at the top), and top search results (list of links with brief information snippets including sponsored content). We analyzed only Google interface-based feedback to be consistent (the first response without link navigation).

Two board-certified physicians (author JL is board certified in pediatrics and pediatric gastroenterology and author FC is board certified in pediatrics) compared the LLM responses and Google Search results to the ACOG FAQ responses and rated the quality of responses using a GRADE (Grading of Recommendations Assessment, Development and Evaluation)-informed scale [6]. We calculated Cohen κ coefficient to measure interrater reliability. We tested the normality (Shapiro-Wilk test) and homoscedasticity (Levene test) of the rater data, followed by the Kruskal-Wallis test to compare the differences in the quality rating among the 3 groups. The pairs of groups were investigated for significant differences by post hoc Dunn test with Bonferroni correction (for multiple comparisons). Analyses used R software (v4.2.1; R Foundation of Statistical Computing) [7].

## Results

ChatGPT differed in the quality of responses against others (mean 3.93, SD 0.27; Table 1). A statistically significant difference in the distribution of scores among the categories was found (*χ*^2^_2_=12.2; *P*=.002; Table 2). ChatGPT demonstrated generally higher quality (more clinically accurate) responses compared to Bard (*Z*=2.143; adjusted *P*=.048) and Google Search (*Z*=3.464; adjusted *P*<.001). There was no difference in the quality of responses between Bard and Google Search (*Z*=1.320; adjusted *P*=.28).

Raters showed perfect agreement for ChatGPT (κ=1, 95% CI 0.85-1.15) and near-perfect agreement for Bard and Google Search (κ=0.92, 95% CI 0.71-1.13). Data were not normally distributed (*P*<.05) and nonhomoscedastic (*F*_2_=4.153; *P*=.02) for each category (ChatGPT, Bard, and Google Search).

**Table 1:** Average quality ratings for ChatGPT, Bard, and Google Search responses to American College of Obstetricians and Gynecologists (ACOG) questions [5].

ACOG postpartum depression frequently asked questions	Average quality ratings^a^		
	ChatGPT	Bard	Google Search
What are baby blues?	4	4	3
Can antidepressants cause side effects?	4	0	3
How is postpartum depression treated?	4	4	4
How long do the baby blues usually last?	4	4	1
If I think I have postpartum depression, when should I see my health care professional?	4	4	1
What are antidepressants?	4	0	3.5
Can antidepressants be passed to my baby through my breast milk?	4	0	3
What are the types of talk therapy?	4	4	3
What can be done to help prevent postpartum depression in women with a history of depression?	3	4	1
What causes postpartum depression?	4	0	1
What happens in talk therapy?	4	4	4
What is postpartum depression?	4	4	4
What support is available to help me cope with postpartum depression?	4	3	1
When does postpartum depression occur?	4	3.5	1
Mean (SD)	3.93 (0.27)	2.75 (1.83)	2.39 (1.3)
Median (IQR)	4 (4-4)	4 (0-4)	3 (1-4)
Mode	4	4	1
Minimum-maximum	3-4	0-4	1-4

^a^GRADE (Grading of Recommendations Assessment, Development and Evaluation)-informed quality assessment scale [6]: 0=no response (the system refused to provide any information), 1=inaccurate response (the system response does not reflect any facts relevant to the corresponding question), 2=clinically inaccurate response (the system response includes facts about the corresponding question but is not clinically relevant), 3=partially clinically accurate response (the system response is accurate and clinically relevant, yet it introduces some risks in terms of misinterpretations and misunderstanding), 4=mostly clinically accurate response (the system response is accurate and clinically relevant, and risk is minimal for misinterpretations and misunderstanding).

**Table 2:** Results of nonparametric test to identify significant differences between categories (Kruskal-Wallis) and post hoc pairwise comparison to determine differing categories (Dunn test).

Test		Value	Adjusted *P* value
**Kruskal-Wallis**			
	Chi-square (*df*)	12.2 (2)	.002^a^
**Dunn Test**			
	ChatGPT vs Bard, *Z* value	2.143	.048^a^
	ChatGPT vs Google Search, *Z* value	3.464	<.001
	Bard vs Google Search, *Z* value	1.320	.28

^a^*P*<.05.

^b^*P*<.001.

## Discussion

This study expands an earlier investigation on chatbot advice for PPD [8], showing that LLMs can provide clinically accurate responses to questions regarding PPD. ChatGPT provides higher-quality responses based on concordance with answers provided in the ACOG FAQ. The quality of Bard responses was high when provided, but its overall score was impacted by no-response answers (which were mostly factual in nature rather than seeking medical advice, eg, “what are antidepressants?”). These responses received the lowest quality score in our rating. Almost all of the responses by Bard and ChatGPT did not provide a source for the information in their responses (only one response included a source). However, many responses recommended consulting a health care provider or mental health professional in some capacity. Google Search results were rated as lower-than-average quality compared to Bard and ChatGPT.

Overall, LLMs showed promise in terms of providing clinically accurate or better-quality responses than Google Search results. This finding is consistent with the prior investigation on the appropriateness of LLM-based medical advice [9]. Our findings should be interpreted carefully considering the following limitations. To start, none of these technologies are built for medical purposes. We included a limited number of standard questions (14 ACOG questions) analyzed within a limited scope (one question per category; no personas, eg, “act like a doctor”; no prompt engineering for exploring different contexts or settings). Future work is needed for a more comprehensive investigation (eg, measuring acceptability and empathy with stakeholders) as well as to develop clinical guidance (frameworks in close collaboration among clinicians, researchers, and developers) to inform the implementation and evaluation of such technologies, ensuring their ability to address PPD-related questions accurately, ethically, and safely [10].

## Appendix

Multimedia Appendix 1 Responses to postpartum depression frequently asked questions.

## Abbreviations

ACOG: American College of Obstetricians and Gynecologists
GRADE: Grading of Recommendations Assessment, Development and Evaluation
LLM: large language model
PPD: postpartum depression

## Data Availability

All data generated or analyzed during this study are included in this published article (Multimedia Appendix 1).
